# Analysis of long non-coding RNA expression profiles identifies novel lncRNA biomarkers in the tumorigenesis and malignant progression of gliomas

**DOI:** 10.18632/oncotarget.18832

**Published:** 2017-06-28

**Authors:** Gang Chen, Yanfei Cao, Lina Zhang, Hongxing Ma, Chao Shen, Jiang Zhao

**Affiliations:** ^1^ Department of Neurosurgery, Daqing Oilfield General Hospital, Daqing 163001, China; ^2^ Department of Clinical Laboratory, Daqing Oilfield General Hospital, Daqing 163001, China; ^3^ Department of Neurosurgery, Shanghai Fourth People's Hospital, Shanghai 200081, China

**Keywords:** biomarkers, gliomas, glioblastoma multiforme, long non-coding RNAs

## Abstract

Long non-coding RNAs have been shown to be associated with cancer development and progression, demonstrating potential applications as novel diagnostic or prognostic molecular markers in clinical management and treatment. However, the functional significance of lncRNAs in the development and malignant progression of gliomas is still unclear and needed to be further explored. we first obtained genome-wide lncRNA expression profiles in a large cohort of patients with gliomas from the Gene Expression Omnibus database using microarray probes repurposing method and investigated the lncRNA expression patterns during the tumorigenesis and malignant progression of gliomas. By using differential expression analysis, we identified a large number of lncRNAs that were associated with the tumorigenesis and malignant progression of gliomas in the training dataset and showed their robustness in the testing dataset. Subsequently, we identified a novel four-lncRNA signature which was closely related to the prognosis of patients with GBM. The prognostic value of this signature was verified in the test set of 80 patients. Functional analysis suggested that the four lncRNAs associated with survival of patients with GBM may be involved in cancer-related biological processes and pathways and their deregulation may lead to GBM tumorigenesis and progression. These novel lncRNA biomarkers will improve our understanding of the molecular mechanisms during the development and progression of glioma and provide novel diagnostic or prognostic markers and therapeutic targets for gliomas.

## INTRODUCTION

Gliomas are the most common type of primary brain tumor, accounting for 80% of all malignant brain tumors and 30% of all brain and central nervous system tumors. Gliomas are further categorized into low-grade gliomas (WHO grade I and II) and high-grade gliomas (WHO grade III and IV) according to their histopathologic characteristics such as cytological atypia, anaplasia, mitotic activity, microvascular proliferation, and necrosis [1]. The median overall survival of patients with gliomas varied significantly across different grades: Patients with low-grade gliomas tended to have a favorable prognosis with a median survival of 11 years to 16.7 years [2], whereas patients with low-grade gliomas often faced poor prognosis with a median survival of 15 months to three years [3]. Recent large-scale omics study has suggested that gliomas is a complex and heterogeneous disease at the genetic and epigenetic levels [4]. Improving our understanding of the molecular mechanisms during the development and progression of glioma is critical for diagnosis, prognosis and therapy of glioma

During the past decades of RNA biology, a novel class of RNAs, termed long noncoding RNAs (lncRNAs), has been identified as an important layer of genome complexity [5]. lncRNAs are generally defined as mRNA-like transcripts ranging in length from 200 nt to ∼100 kilobases lacking significant protein-coding capacity [6]. There are a lot of evidence that lncRNAs are involved in the regulation at chromatin organization, transcriptional, and post-transcriptional levels [7]. With the advent of advanced sequencing technologies and transcriptome research, a number of differentially expressed lncRNAs have been identified to be associated with cancer development and progression, demonstrating potential applications as novel diagnostic or prognostic molecular markers in clinical management and treatment [8]. In accordance with their significant roles in other human cancers, recent some studies have demonstrated a significant association between lncRNA expression and gliomas [9–11]. Although the prognostic roles of lncRNAs have been investigated preliminarily in several studies [12, 13], the functional significance of lncRNAs in the development and malignant progression of gliomas are still unclear and needed to be further explored.

The purpose of this study is to identify specific lncRNA markers significantly associated with the tumorigenesis and malignant progression of gliomas by studying differential lncRNA expression pattern in glioma and normal brain tissue or in different WHO grades in a large cohort of patients with gliomas from the Gene Expression Omnibus (GEO), and identify a lncRNA signature which could act as a prognostic predictor for patients with glioblastoma multiforme (GBM).

## RESULTS

### Identification and validation of lncRNAs associated with glioma development

**Figure 1:**
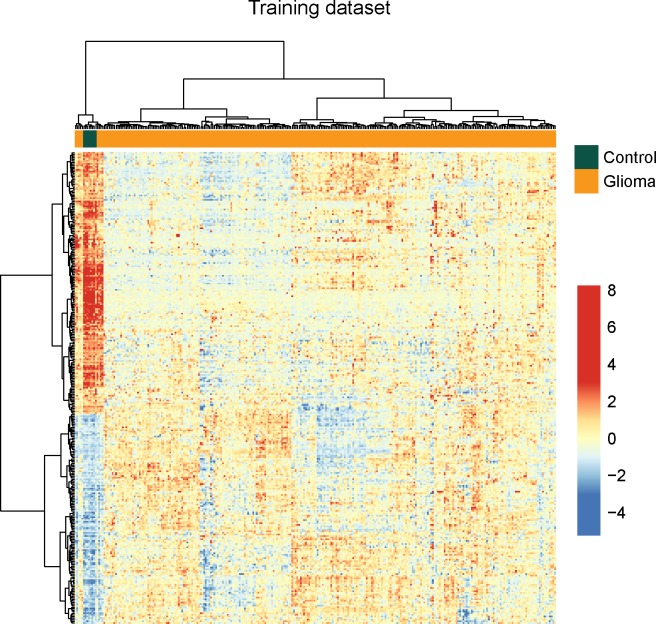
The heatmap of hierarchical clustering of differentially expressed lncRNAs between glioma samples and normal brain tissues in the training dataset

To identify potential lncRNAs associated with glioma development, we performed differential expression analysis for lncRNAs between normal brain tissues (n=8) and glioma samples (n=276) using significant analysis of microarray (SAM) method in the training dataset. In total, we identified 299 lncRNAs that are differentially expressed in normal brain tissues compared to glioma samples using SAM with an adjusted P-value <0.01 after Benjamini & Hochberg correction and fold change > 2.0 (or <0.5). Of these, 133 lncRNAs were over-expressed and 166 lncRNAs were down-regulated in glioma samples compared to normal brain tissues. Hierarchical clustering of all samples in the training dataset according to the expression value of these 299 differentially expressed lncRNAs based on centred Pearson correlation clearly separated glioma samples from normal brain tissues (Figure 1). Statistical analysis showed that two major sample clusters were highly correlated with the sample status (p<0.001, Chi-square test). As shown in Figure 1, the left sample cluster contained all the normal samples and 9 glioma samples, and the right sample cluster contained 267 out of glioma samples. Results of hierarchical clustering suggested these 299 differentially expressed lncRNAs can distinguish glioma samples from normal brain tissues with 96.83% accuracy.

**Figure 2:**
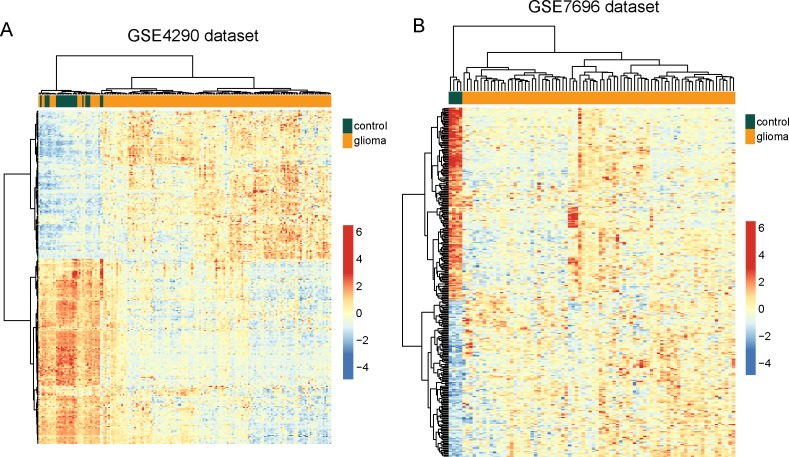
Validation of differentially expressed lncRNAs between glioma samples and normal brain tissues in the two testing datasets **(A)** The heatmap of hierarchical clustering of differentially expressed lncRNAs between glioma samples and normal brain tissues in the GSE4290 dataset. **(B)** The heatmap of hierarchical clustering of differentially expressed lncRNAs between glioma samples and normal brain tissues in the GSE7696 dataset.

To validate the predictive value of these 299 differentially expressed lncRNAs, hierarchical clustering of these 299 differentially expressed lncRNAs were applied to all samples in the GSE4290 and GSE7696 datasets. These two independent datasets confirmed the predictive ability of these 299 differentially expressed lncRNAs in distinguishing glioma samples from normal brain tissues. As shown in Figure 1B, two distinctive sample clusters were obtained by hierarchical clustering analysis for GSE4290 samples. Only 19 samples (17 glioma samples and 2 normal brain tissues) were misclassified by the clustering analysis. Cluster 1 consisted of 38 samples, including 17 glioma samples and 21 normal brain tissues, whereas cluster 2 consisted of 142 samples, including 140 glioma samples and 2 normal brain tissues, which achieved a high prediction accuracy of 89.44%. The statistical result suggested that two sample clusters grouped by these 299 lncRNAs were significantly correlated with sample disease status (p<0.001, Chi-square test) (Figure 2A). Similar results were observed when the GSE7696 dataset was analyzed separately. As shown in Figure 2B, one group contained all the normal brain samples (n=4) and the second group contained all glioma samples (n=80). Thus, the above results demonstrated the overall reliability of the predictive value of these dysregulated lncRNAs, making them as good candidates for glioma-specific markers.

### Identification and validation of lncRNAs in malignant progression of glioma

**Figure 3:**
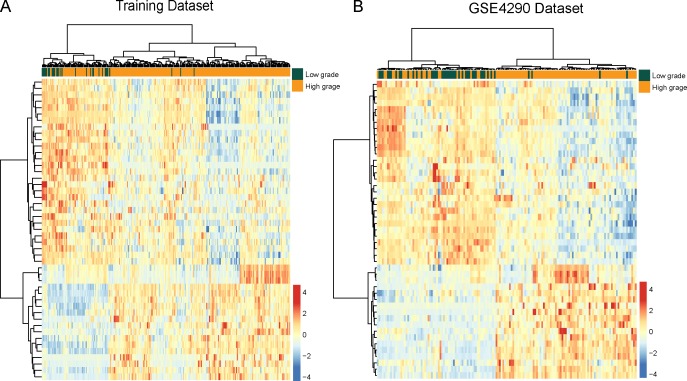
The heatmap of hierarchical clustering of differentially expressed lncRNAs between low-grade gliomas and high-grade gliomas **(A)** The heatmap of hierarchical clustering of differentially expressed lncRNAs between low-grade gliomas and high-grade gliomas in the training dataset. **(B)** The heatmap of hierarchical clustering of differentially expressed lncRNAs between glioma samples and normal brain tissues in the GSE4290 dataset.

To identify potential lncRNAs associated with malignant progression of glioma, we performed differential expression analysis for lncRNAs between low-grade gliomas (grade I and II, n=32) and high-grade gliomas (grade III and IV, n=244) using SAM method in the training dataset. In total, we identified 47 lncRNAs that are differentially expressed between low-grade gliomas and high-grade gliomas using SAM with an adjusted P-value <0.01 after Benjamini & Hochberg correction and fold change > 2.0 (or <0.5). Of these, 18 lncRNAs were over-expressed and 29 lncRNAs were down-regulated in high-grade gliomas compared to low-grade gliomas. We clustered the glioma samples in the training dataset according to the expression values of 47 differentially expressed lncRNAs between low-grade gliomas and high-grade gliomas and obtained two distinctive sample clusters: cluster 1 containing 28 out of 32 low-grade gliomas and 198 of 244 high-grade gliomas with an 88.41% accuracy (Figure 3A). Statistical analysis showed that two major sample clusters were highly correlated with cancer grades (p<0.001, Chi-square test).

For the predicted progression-related lncRNAs markers, we have examined their expression patterns with respect to grades in the independent GSE4290 dataset by using hierarchical clustering and Chi-square test. As shown in Figure 3B, similar results were observed where two major sample clusters was identified: Cluster 1 consisted of 70 samples, including 41 low-grade gliomas and 29 high-grade gliomas, whereas cluster 2 consisted of 83 samples, including 4 low-grade gliomas and 79 high-grade gliomas, which achieved a high prediction accuracy of 78.43%. The statistical result suggested that two sample clusters grouped by these 47 lncRNAs were significantly correlated with cancer grades (p<0.001, Chi-square test).

### Identification of lncRNA biomarkers associated with outcome of patients with GBM in the training dataset

**Figure 4:**
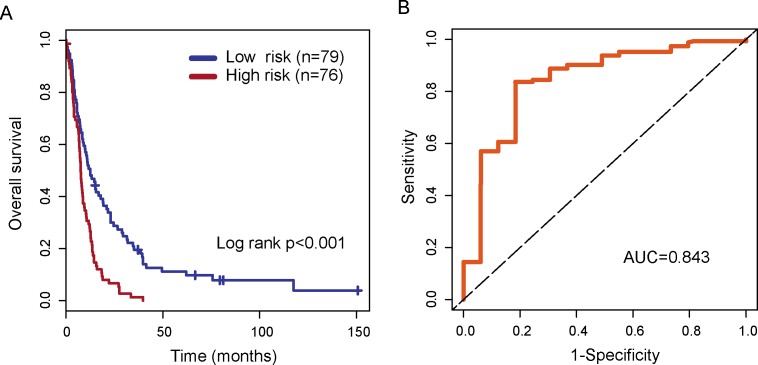
The performance of the four-lncRNA signature in the training dataset **(A)** Kaplan-Meier survival curves of overall survival between high-risk group and low-risk group in the training dataset. **(B)** Time-dependent receiver operating characteristics (ROC) curves in the training set.

To identify novel lncRNA biomarkers associated with the outcome of patients with glioblastoma multiforme, we used univariate Cox proportional hazard regression to each of 324 lncRNAs associated with the development and malignant progression of glioma and identified 78 candidate lncRNAs significantly associated with the outcome of patients with GBM in the training dataset. Then a multivariable Cox regression analysis was performed for all 78 candidate lncRNAs and identified four independent lncRNA biomarkers (*AK098425*, *AL833059*, *AK056155* and *CR613436*) significantly associated with the outcome of patients with GBM. We then used these four lncRNA biomarkers to construct an expression signature by the risk score method as the classifier for prognosis prediction. This four-lncRNA expression signature was defined as a linear combination of the expression levels of the four lncRNA biomarkers and the multivariate Cox regression coefficient as the weight as follows: Risk score=(0.229*expression value of *AK098425*)+(−0.210*expression value of *AL833059*)+ (0.257*expression value of *AK056155*)+(0.291*expression value of *CR613436*). The risk score was calculated for each patient in the training dataset. Then patients of training dataset were assigned into the high-risk group (n=76) and low-risk group (n=79) using the median risk score as risk cutoff value (0.27). Survival analysis suggested that there is a significant difference in overall survival between high-risk group and low-risk group (p<0.001, log-rank test) (Figure 4A). Patients in the high-risk group had significantly shorter overall survival than those in the low-risk group (median survival 7.68 months vs. 12.60 months). The three year-survival rate of patients is 1.3% in the high-risk group, which is significantly lower than that in the low-risk group (19.6%). Time-dependent ROC curves were used to assess the prognostic power of the four-lncRNA signature. The AUC for the four-lncRNA signature prognostic model was 0.843 at 36 months of overall survival (Figure 4B).

### Validation of lncRNA biomarkers associated with outcome of patients with GBM in the independent testing dataset

**Figure 5:**
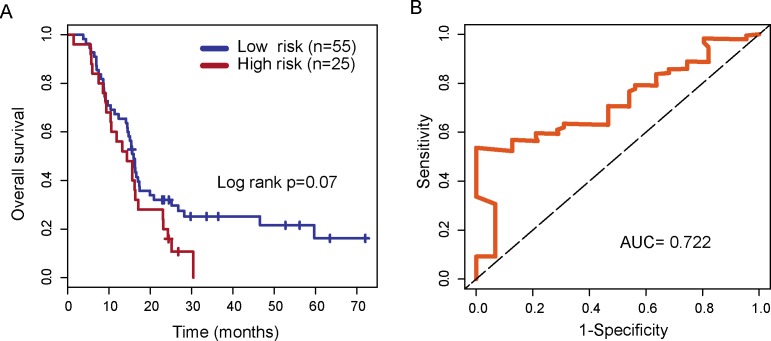
Validation of the four-lncRNA signature in the testing dataset **(A)** Kaplan-Meier survival curves of overall survival between high-risk group and low-risk group in the testing dataset. **(B)** Time-dependent receiver operating characteristics (ROC) curves in the testing dataset.

To evaluate the robustness of four lncRNA biomarkers, the four-lncRNA signature was further validated in another independent testing dataset (GSE7696 dataset). By using the same risk score model and cutoff value from the training dataset, 80 patients in the GSE7696 dataset were divided into the high-risk group (n=25) and low-risk group (n=55). Inconsistent with the findings described above, patients in high-risk group and low-risk group showed marginally significantly different overall survival (p=0.07, log-rank test) (Figure 5A). Patients in the high-risk group had significantly shorter overall survival than those in the low-risk group (median survival 14.3 months vs. 16 months). The three year-survival rate of patients is 0 % in the high-risk group, which is significantly lower than that in the low-risk group (25%). The AUC of time-dependent ROC curves for the four-lncRNA signature prognostic model was 0.722 at 36 months of overall survival (Figure 5B).

### Functional analysis of the four-lncRNA signature

**Figure 6:**
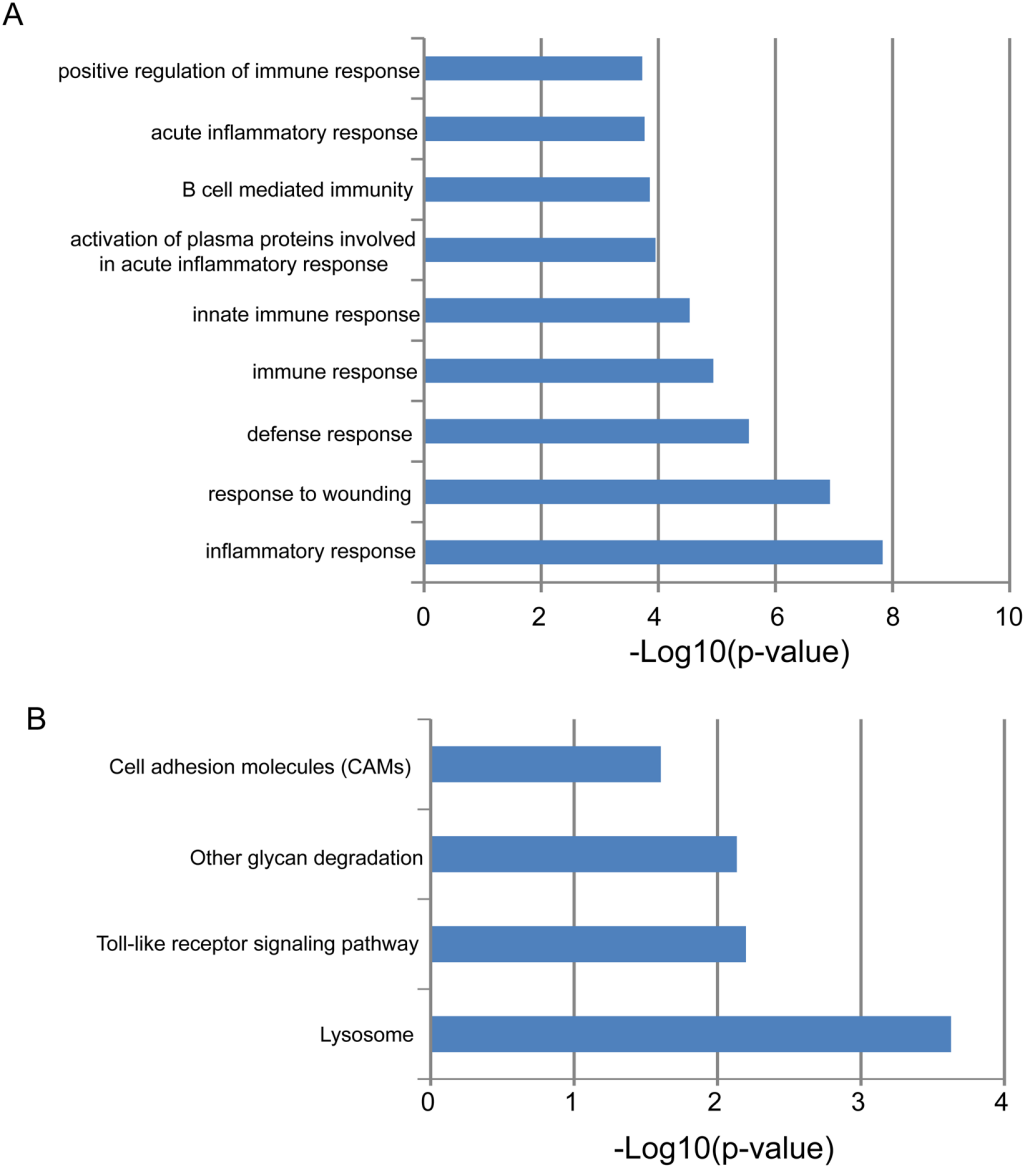
Functional maps of the four-lncRNA signature **(A)** Enriched GO terms. **(B)** Enriched KEGG pathways.

In order to gain an initial understanding of functional roles of this four-lncRNA signature, we first calculated the expression correlation between one of four lncRNAs and protein-coding genes in the training dataset and identified 664 protein-coding genes that are positively correlated with at least one of the four prognostic lncRNAs (top 1%). Then we performed functional enrichment analysis for protein-coding genes at GO and KEGG levels to infer lncRNA function. Results of GO analysis suggested that these 664 protein-coding genes enriched significantly in immune-related biological processes, including inflammatory response, response to wounding, inflammatory response, response to wounding, defense response, immune response, innate immune response, activation of plasma proteins involved in acute inflammatory response, B cell mediated immunity, acute inflammatory response and positive regulation of immune response (Figure 6A). Results of KEGG analysis suggested that these 664 protein-coding genes enriched significantly in four biological pathways (Figure 6B), including Lysosome, Toll-like receptor signaling pathway, Other glycan degradation and Cell adhesion molecules (CAMs).

## DISCUSSION

Although traditional histopathologic classification is well established based on histological features, histological classifications did not well reflect clinical characteristics and prognosis of patients with grade II-III glioma and glioblastoma (grade IV glioma) [14, 15]. With the advent of advanced sequencing technologies and transcriptome analysis, recent molecular characterization studies have suggested that gliomas are characterized by not only heterogeneous histological features but also heterogeneous molecular features. For example, previous gene expression profiles-based studies have demonstrated the presence of three distinct gene expression signatures of gliomas [16]. A recent study performed by Eckel-Passow also proposed a new molecular classification of glioma into three molecular subtypes: IDH wild-type cases, IDH mutant-codel and IDH-mutant-non-codel [17]. Although these research efforts have resulted in a better understanding of the molecular basis of glioma formation as well as the genetic alterations and produce clinical application for glioma management, previous studies only focused on molecular characterization at the mRNA or miRNA levels.

More recently, an extensive amount of functional non-coding RNAs has been discovered by experimental or computational approaches. Many of them have been proved to play important roles in both oncogenic and tumor suppressive pathways [18]. Aberrant lncRNA expression has been widely observed in various human cancers by transcriptional profiling analysis, highlighting their hallmark feature in cancer [19]. Moreover, lncRNA tended to be expressed in a more type-specific or tissue-specific manner, and are detectable in the blood, sputum, and urine of cancer patients [20–22], making them as novel ideal candidate biomarkers for early diagnosis and prognosis prediction in cancer. The diagnostic or prognostic roles of lncRNAs have been explored and investigated in some cancers, including gliomas [23–33]. However, the functional significance of lncRNAs in the development and malignant progression of gliomas are still unclear and needed to be further explored.

In our study, we first obtained genome-wide lncRNA expression profiles in a large cohort of patients with gliomas from the Gene Expression Omnibus database using microarray probes repurposing method, and investigated the functional roles of lncRNAs in the tumorigenesis and malignant progression of gliomas by examining expression pattern of lncRNAs in glioma and normal brain tissue or different WHO grades. By using differential expression analysis, we identified a large number of lncRNAs that were associated with the tumorigenesis and malignant progression of gliomas in the training dataset and showed their robustness in the testing dataset. Our results demonstrated that dysregulated lncRNA expression played important roles in the tumorigenesis and malignant progression of gliomas. Hierarchical clustering analysis revealed that lncRNA expression pattern was significantly correlated with disease status. Finally, we identified a novel four-lncRNA signature which was closely related to the prognosis of patients with GBM. The prognostic value of this signature was verified in the test set of 80 patients.

In recent years, an increasing number of lncRNAs have been identified. However, the functional study of lncRNAs is relatively slow and only a fraction of lncRNAs was well functionally characterized. It has been shown that lncRNA function could be inferred by studying the functional roles of protein-coding genes that are coexpressed with lncRNAs. Therefore, we performed a preliminary functional study for these four prognostic lncRNAs by performing functional enrichment analysis for co-expressed protein-coding genes. Results of functional enrichment analysis suggested that genes co-expressed with four prognostic lncRNAs tended to be clustered most significantly in nine immune-related GO biological processes and four KEGG biological pathways. Previous studies have demonstrated the close association between immune response and outcome in GBM [34, 35]. Lysosomes are membrane-bound intracellular organelles and have been proven to be involved in cell death and cancer [36]. A recent study performed by Giatromanolaki et al. found that lysosomal markers were also intensively upregulated in glioblastomas and may be a new therapeutic target [37]. Expression of some cell adhesion molecules, such as ICAM-1 and LFA-3, is dysregulated in GBM compared with normal brain tissue and can serve as a novel marker for brain tumor detection and perhaps therapy [38, 39]. Thus it is a plausible inference that the four lncRNAs associated with survival of patients with GBM may be involved in cancer-related biological processes and pathways and their deregulation may lead to GBM tumorigenesis and progress. However, further biological experiments should be conducted to investigate the biological roles in GBM.

In conclusion, we investigated the lncRNA expression patterns during the tumorigenesis and malignant progression of gliomas and their effects on patients’ disease status. Our study has shown that the lncRNA expression pattern is altered during the tumorigenesis and malignant progression of gliomas which can serve as novel molecular markers for diagnosis and prognosis prediction. Using lncRNA expression profiling in Cox proportional hazard regression analysis, we developed a novel four-lncRNA signature that accurately predicts survival in patients with GBM. These novel lncRNA biomarkers will improve our understanding of the molecular mechanisms during the development and progression of glioma and provide novel diagnosis or prognosis markers and therapeutic targets for gliomas.

## MATERIALS AND METHODS

### Patients and samples

Patients and normal samples used in this study were from three independent datasets in the Gene Expression Omnibus database (http://www.ncbi.nlm.nih.gov/geo/). The dataset GSE16011 (https://www.ncbi.nlm.nih.gov/geo/query/acc.cgi?acc=GSE16011) was selected as our training dataset because it is the largest dataset. Another two datasets GSE4290 (https://www.ncbi.nlm.nih.gov/geo/query/acc.cgi?acc=GSE4290) and GSE7696 (https://www.ncbi.nlm.nih.gov/geo/query/acc.cgi?acc=GSE7696) were used as testing datasets. The training dataset contained 276 glioma samples with overall survival information, including 8 grade I sample, 24 grade II samples, 85 grade III samples and 159 grade IV samples, as well as 8 non-tumoral brain tissue controls. The testing GSE4290 dataset contained 157 glioma samples without overall survival information, including 45 grade II samples, 31 grade III samples and 77 grade IV samples, as well as 23 non-tumoral brain tissue controls. Another testing GSE7696 dataset contained 80 glioblastoma multiforme (GBM) samples with overall survival information and 4 non-tumoral brain tissue controls.

### Acquisition and analysis of lncRNA expression profiles

The raw CEL files of these three datasets on the Affymetrix HG-U133 Plus 2.0 platform were downloaded from GEO. These raw CEL files were processed and normalized using the Robust Multichip Average (RMA) algorithm for background adjustment [64] and log-transformed (base 2). LncRNA expression profiles of CC patients in this study were obtained by repurposing the probes in the HG-U133_Plus_2.0 array according to Zhang's study [40]. Briefly, the Affymetrix HG-U133 Plus 2.0 probe set ID was mapped to the NetAffx Annotation Files. The probe sets that were assigned with a RefSeq transcript ID and/or Ensembl gene ID in the NetAffx annotations were extracted. For the probe sets with RefSeq IDs, only those probe sets labeled as“NR_”were retained. Finally, 3475 probes (probe sets) representing 2466 lncRNAs were obtained by remapping their RefSeq IDs and Ensembl IDs to the annotation of lncRNAs from GENECODE.

Significance analysis of microarrays (SAM) was applied to identify differentially expressed lncRNAs between normal brain tissues and glioma samples, and between low-grade gliomas (grade I and II) and high-grade gliomas (grade III and IV). Those lncRNAs with FDR ≤ 0.01(Benjamini and Hochberg's multiple test adjustment) and fold change > 2.0 (or <0.5) from SAM analysis were identified as differentially expressed lncRNAs. Unsupervised hierarchical clustering was used to investigate the effectiveness of lncRNA biomarkers in distinguishing normal brain tissues and glioma samples or low-grade gliomas (grade I and II) and high-grade gliomas (grade III and IV), and the Chi-square test was used to test the significance of the association between sample status and lncRNA biomarkers.

### Statistical analysis

To identify novel lncRNAs significantly associated with overall survival of patients with GBM, the univariate and multivariate Cox regression analysis were performed to evaluate the association between each of dysregulated lncRNAs and patient's overall survival. Then a lncRNA signature was developed as a linear combination of the expression value of lncRNAs weighted by their respective multivariate Cox regression coefficients. According to the lncRNA signature, patients were classified into high-risk group and low-risk group using the median risk score from the training dataset as the cutoff. Kaplan-Meier survival curves and log-rank tests were used to assess the differences in survival time between the predicted early-stage-like group and advanced-stage-like patients. Time-dependent ROC analysis was performed to compare the sensitivity and specificity of the survival prediction based on the lncRNA risk score. All analyses were performed using R/Bioconductor.
